# Improving reproducibility of photocatalytic reactions—how to facilitate broad application of new methods

**DOI:** 10.1038/s41467-023-44362-0

**Published:** 2024-01-05

**Authors:** Santiago Cañellas, Manuel Nuño, Elisabeth Speckmeier

**Affiliations:** 1 Chemical Capabilities, Analytical & Purification, Global Discovery Chemistry, Janssen Research and Development, Janssen-Cilag, S.A., E-45007 Toledo, Spain; 2 Vapourtec Ltd. Park Farm Business Centre, Fornham St Genevieve, Bury St Edmunds, Suffolk, IP28 6TS United Kingdom; 3 Sanofi, R&D, Integrated Drug Discovery, Industriepark Höchst, 65926 Frankfurt am Main, Germany

**Keywords:** Photocatalysis, Analytical chemistry

## Abstract

Photocatalysis, as a powerful tool for synthetic transformations, has the potential to impact industrial applications. In this commentary, we discuss the challenges and requirements with respect to reproducing published photocatalytic reactions in the (life science) industry.

In the past few decades, photocatalysis has emerged as an exceptionally powerful tool in synthetic organic chemistry due to its capacity to access novel chemical space by enabling previously unfeasible bond formations. In a relatively short period, these methodologies have been applied in subfields of synthetic chemistry such as C-H activation, C-C and C-Heteroatom bond forming reactions, the synthesis of heterocycles, isotope labeling or peptide functionalization, to name but a few^1–3^.

These subfields of synthetic chemistry hold tremendous potential to impact our society through their application in the discovery of pharmaceutical or agrochemical ingredients as well as new materials^4,5^. Nonetheless, there are opportunities to shorten the gap between the initial discovery and its use across laboratories, which would enhance further the impact of these important innovations^6^. In this commentary, we would like to analyze what lengthens the timelines from discovery to broad application and highlight opportunities to shorten this timeline.

## Publication standards—key to improving reproducibility

Using only a light source and a vessel, photocatalytic reaction setups are easily accessible in academic and industrial environments. This simplicity, together with the increasing commoditization of high-power UV and visible light sources (e.g., LEDs), made reactor design a white canvas for ingenuity and enabled numerous different approaches to photocatalytic applications. However, with such an array of different experimental setups^7^, precise reporting of the reaction parameters becomes critical to transpose reaction conditions between different configurations and/or reactors and to ensure reproducibility.

A descriptive reporting of critical parameters, including details regarding the light source and chosen setup, would improve the success rate when reproducing photochemical reactions across various laboratories and should be part of every publication in addition to the chemistry-related information. Missing data about differences in photon flux, reaction temperature, atmosphere, mass transfer (shaking/stirring/mixing) and solubility are primary reasons for the limited reproducibility of published reactions^8,9^.

With the light source as the central element of every reactor, a reasonable characterization providing information about the spectral output (or max peak & full width at half maximum (FWHM) for LEDs) and the intensity (W/m^2^) is crucial to transfer and reproduce reaction conditions but is often absent in published protocols. Furthermore, as light sources are intrinsically inefficient, they emit not only photons but also heat. Besides affecting the lifetime and runtime-dependent intensity of the lamp itself, this heat is radiated to the reaction mixture, increasing its temperature. In addition, reaction-internal processes such as relaxation from higher vibronic states to the lowest vibrational level (Kasha’s rule) or the ground state (internal conversion) of the excited photocatalyst can cause a significant rise in the temperature of a reaction mixture. This can lead to numerous critical processes like unproductive thermal pathways, a change in reaction kinetics, solvent evaporation, or an increased solubility of substances in heterogeneous mixtures, resulting in different outcomes of a photocatalytic reaction. A description of utilized cooling systems for lamps and reaction vessels (e.g., fans) is a rough indication. Still, only an accurate temperature measurement of the reaction mixture itself exactly determines this parameter in experimental setups and increases potential reproducibility^8^.

Depending on the setup or type of reactor, a uniform irradiation field may not be achieved, causing a distribution of over- and under-irradiated substrate, which in turn increases the difficulty of accurately characterizing photochemical kinetics. The reactor material and its geometry can significantly impact how many photons reach the substrate or catalyst due to light absorbance, transmission processes or reflection^9^. In addition, the light intensity also attenuates as it travels through air or any other medium, proportionally to the square of the distance between the source and the reactor, causing a dependency between the reaction outcome and the distance between the vessel and the light source. According to the Lambert-Beer rule, the photonic flux decreases exponentially with the path length in a medium. Due to the often high extinction coefficients of photocatalysts used in visible light photocatalysis, light penetration in such reactions often reaches only the first few millimeters of the reaction mixture in the vessel^10^. Due to this effect, parameters such as mass transfer (stirring/shaking/mixing) and the geometry of a reaction vessel or the diameter in a flow system can be crucial for reproducibility but are often omitted in descriptions^4,11,12^.

## High-throughput photocatalysis

Nowadays, high-throughput chemistry is of utmost importance in any laboratory for condition screening (high-throughput experimentation (HTE)), reaction optimization (design of experiments (DoE)), and parallel synthesis, as well as the generation of reactivity predictive artificial intelligence/machine learning (AI-ML) models^13,14^. To realize these approaches for photochemical transformations, multiple research groups and companies pursue the development of parallel photoreactors^15,16^.

Considering the reproducibility and robustness issues presented herein, developing and identifying reliable parallel photoreactors has become an essential technological challenge over the last years. The uniformity of all discussed parameters for every single position within the reactor is crucial to make decisions based on a robust data set. To meet these criteria, homogeneous irradiation across the array, constant temperature control of every vessel (or well), efficient shaking/stirring to avoid mass transfer limitations, and adequate plate sealing is necessary.

As reported recently by different groups, uniformity in parallel photoreactors can be challenging to achieve, and setups have to be carefully evaluated. A simple experiment to evaluate the robustness and reproducibility of a parallel photoreactor and its ability to provide homogeneous reaction conditions is carrying out the same reaction across a plate and analyzing the reaction for every position^16,17^. It is also advised to analyze the reaction at moderate conversions to identify potential kinetic differences^18^. Discrepancies in the outcome can flag underlying problems, and trends observed can inform potential solutions. Other measurements that provide valuable information about the performance of a photoreactor are the internal photon flux and internal temperature during the course of a reaction.

Reporting these control experiments alongside the technical specifications of the parallel photoreactor would not only be of added value for the scientific community, but it would contribute to a more robust data set and higher reproducibility.

## Photocatalysis in continuous flow

Enabling technologies such as continuous flow can offer more intense and uniform irradiation of the reaction mixture compared to batch reactors. By reducing the distance between the reactor and the light source, as well as shortening the irradiation path length in the reactor itself, a more consistent distribution of photons is achieved^19^. Furthermore, the characterization of photochemical kinetics can be done more precisely, as flow chemistry allows for a linear scale^20^.

Despite these advantages, reproducibility in continuous flow can still be challenging to achieve. As the molar ratio photon over substrate or catalyst defines its level of success, the collection of the products can be a critical parameter, as the fraction collection outside a window of steady-state conditions can cause hugely variable results due to a change of the molar ratio by dilution.

## Applicability of photocatalyzed methods in life science industry

While the reproducibility of photochemical reactions mainly relies on the parameters discussed herein, the applicability of photocatalytic strategies in the pharmaceutical and agrochemical industry is additionally affected by other factors. Unlike an exact reproduction, applicability here does not mean repeating the reaction with the exact same substrates but applying a method to generate analogs with similar but distinct properties. What researchers in life science fields are most often aiming for is to quickly generate answers by building highly functionalized compounds. However, the use of new methods has to match the pace of fast-moving projects. With almost countless published manuscripts describing novel reaction concepts, several criteria dictate to what extent a reaction concept gets interpreted as valuable and is worth investigating.

The commercialization of catalysts or ligands requiring a complex multi-step synthesis route is highly desirable to increase the likelihood of testing this chemistry across laboratories, thus shortening the timeline for them to have a broad impact. In cases where used reagents and starting materials are not commercially available, the concept has to demonstrate a substantial impact or improvement over known methodologies in order to be considered for application.

A similar situation applies to the usage of bespoke reaction setups or non-standardized LED lamps with uncommon wavelengths or optical powers. An exact reproduction of a setup using homemade electronic parts (e.g., self-soldered LED arrays) is time-consuming and often accompanied by additional bureaucracy since regulations must be adhered to (e.g., a CE certificate is necessary to operate an electronic device within Europe).

In addition to the commercial availability of reagents and setups, scientists in industry are much more likely to engage in testing new methodologies showcasing a diverse substrate scope and limitations of the methodology, including unsuccessful or low-yielding substrates. For example, in life-science projects, the target compounds are often hydrophilic and highly functionalized; thus, seeing these types of moieties can boost confidence in a given method, help overcome the initial barrier to adopt it, and decrease the chances of being overlooked by other scientists, who would then have a more informed decision-making process.

Besides an informative substrate scope, the effects of different potential contaminations or additives and the exact procedure, including all necessary parameters, are of great help for a successful wider use of a published reaction. For example, it is crucial to know if air exposition can inhibit a reaction, if traces of water are problematic or if the presence of specific functional groups is detrimental in order to apply a reaction not only in a single batch but in different reactors, parallel reaction setups or upscaling processes^21–24^. For this purpose, approaches such as sensitivity assessments are helpful to make a data-driven decision on applying such methods.

Furthermore, the reporting of additional data (pictures) regarding potential handling pitfalls, e.g., a picture showing the reaction setup or the color of a complex, could be helpful for the successful application of published photochemical methods by others.

## Concluding remarks

In summary, we believe that there are multiple reasons for the often large gap between the emergence of photochemical transformations and their broad application across laboratories, but the lack of reproducibility is likely the most relevant one of them. In this regard, the development and use of standardized setups, as well as reporting critical parameters, holds the potential to overcome these issues. These two key aspects, together with the considerations discussed above, can increase reproducibility and applicability, boosting the impact of these great initial discoveries.
